# Formation, Identification, and Occurrence of the Furan-Containing β-Carboline Flazin Derived from l-Tryptophan and Carbohydrates

**DOI:** 10.1021/acs.jafc.3c07773

**Published:** 2024-03-12

**Authors:** Tomás Herraiz, Antonio Salgado

**Affiliations:** † Instituto de Ciencia y Tecnología de Alimentos y Nutrición (ICTAN-CSIC), Spanish National Research Council (CSIC), José Antonio Novais 6, Ciudad Universitaria, Madrid 28040, Spain; ‡ Centro de Espectroscopía de RMN (CERMN), Universidad de Alcalá (UAH), Campus Universitario Ctra. Madrid-Barcelona km 33.6, Alcalá de Henares, Madrid 28805, Spain

**Keywords:** flazin, β-carbolines, tryptophan, 3-deoxyglucosone, fructose, glucose, sucrose, Maillard reaction, foods

## Abstract

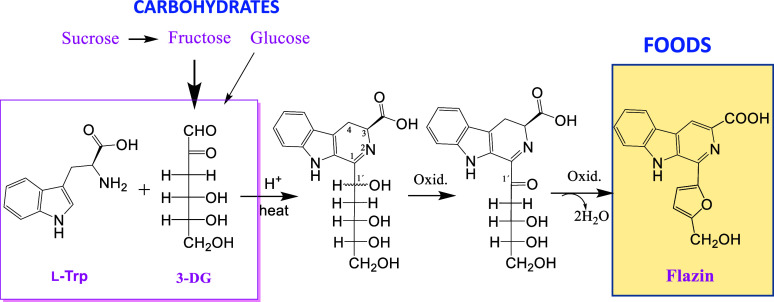

β-Carbolines (βCs) are bioactive indole alkaloids found in foods and in vivo. This work describes the identification, formation, and occurrence in foods of the βC with a furan moiety flazin (1-[5-(hydroxymethyl)furan-2-yl]-9*H*-pyrido[3,4-*b*]indole-3-carboxylic acid). Flazin was formed by the reaction of l-tryptophan with 3-deoxyglucosone but not with 5-hydroxymethylfurfural. Its formation was favored in acidic conditions and heating (70–110 °C). The proposed mechanism of formation occurs through the formation of intermediates 3,4-dihydro-β-carboline-3-carboxylic acid (imines), followed by the oxidation to C=O in the carbohydrate chain and aromatization to βC ring with subsequent dehydration steps and cyclization to afford the furan moiety. Flazin is generated in the reactions of tryptophan with carbohydrates. Its formation from fructose was higher than from glucose, whereas sucrose gave flazin under acidic conditions and heating owing to hydrolysis. Flazin was identified in foods by HPLC-MS, and its content was determined by HPLC-fluorescence. It occurred in numerous processed foods, such as tomato products, including crushed tomato puree, fried tomato, ketchup, tomato juices, and jams, but also in soy sauce, beer, balsamic vinegar, fruit juices, dried fruits, fried onions, and honey. Their concentrations ranged from not detected to 22.3 μg/mL, with the highest mean levels found in tomato concentrate (13.9 μg/g) and soy sauce (9.4 μg/mL). Flazin was formed during the heating process, as shown in fresh tomato juice and crushed tomatoes. These results indicate that flazin is widely present in foods and is daily uptaken in the diet.

## 1 Introduction

**Figure 1:**
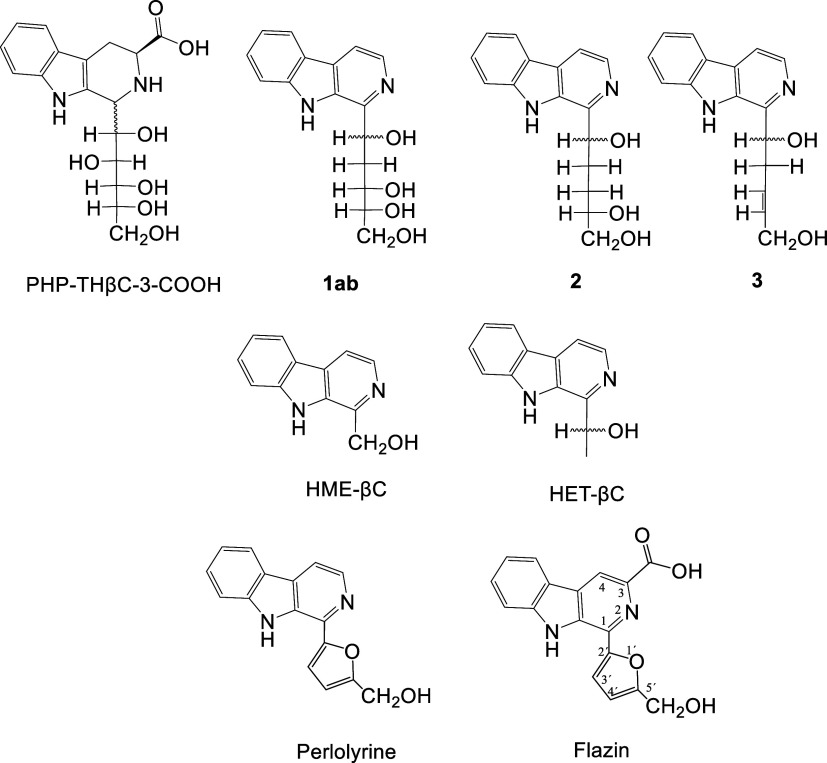
Structures of THβCs and βCs derived from carbohydrates, α-dicarbonyl compounds, and furan-containing βCs.

β-Carboline (βC) alkaloids are bioactive compounds with antitumoral, antimicrobial, antiparasitic, and antioxidant properties, among others. They inhibit key enzymes such as monoamine oxidase (MAO) and kinases and interact with receptors of the human central nervous system (CNS).^1−9^ These alkaloids exert antidepressant and behavioral effects, which are associated with changes in neurotransmitters and MAO inhibition^10−15^ and may also show neuroprotective and antioxidant effects.^16−19^ On the other hand, βC alkaloids have attracted toxicological interest because they can be bioactivated to give endogenous neurotoxins (β-carbolinium cations),^3^ bind to DNA, and are comutagenic in the presence of aromatic amines.^17,18^ The βC alkaloids are naturally occurring compounds that appear in foods and in vivo.^1−9^ These alkaloids are divided into tetrahydro-β-carbolines (THβCs) and aromatic βCs. THβCs form from indoleethylamines and aldehydes, or α-keto acids, by the Pictet–Spengler reaction.^1^ Thus, tryptophan affords tetrahydro-β-carboline-3-carboxylic acids (THβC-3-COOH), which have been largely studied in foods.^2,19−21^ The reaction of tryptophan with glucose produces pentahydroxypentyl-tetrahydro-β-carboline-3-carboxylic acid (PHP-THβC-3-COOH)^22,23^ (Figure 1). This THβC was reported in processed tomato products, fruit juices, and jams,^22^ and in human urine.^24,25^ THβCs afford aromatic βCs through oxidation.^26,27^ Norharman and harman, which are two main aromatic βC compounds, come from the oxidative decarboxylation of THβC-3-COOH in foods.^26,28^ Aromatic βCs arising from carbohydrate (**1ab-3**) (Figure 1) are found in foods at concentrations reaching up to 11.4 μg/g and also in natural products.^1,29−31^ Those are produced in the reactions of tryptophan and fructose, sucrose, and glucose to a lesser extent;^29^ however, they do not form by oxidation of THβCs.^29^ Carbohydrate-derived βCs (**1ab-3**) have been shown to occur by the reaction of 3-deoxyglucosone with tryptophan.^29,32^ It does not follow a classical Pictet–Spengler reaction because it goes through the formation of dihydro-βC derivatives. Following this mechanism of reaction, new βC alkaloids derived from α-dicarbonyls (glyoxal, methylglyoxal, and 3-deoxyglucosone) have been described in foods^32^ (Figure 1).

The βCs containing a furan moiety perlolyrine and flazin have been previously found in natural products^33−35^ and soy sauce^35,36^ (Figure 1). These βCs are bioactive substances. They are chemopreventive agents inducing phase II enzymes.^36^ Perlolyrine is an antiproliferative agent against tumor cells in vitro at micromolar levels.^37^ Flazin is a promising activator of Keap1-Nrf2 involved in antioxidant protection at 250–500 μM levels in vitro,^38^ and it is an active compound against lipid droplet accumulation in hepatocytes.^39^ However, very little is known about the significance, formation, and presence of these furan βCs in foods. In a recent study, we have reported the presence and formation of perlolyrine in foods.^40^ The aim of this study was to describe the formation and occurrence of flazin in foods. The factors involved in the formation of flazin are described for the first time, and the formation mechanism is highlighted as arising from 3-deoxyglucosone, a product of carbohydrate degradation. Flazin was produced in the reaction of tryptophan with carbohydrates. Flazin was identified and analyzed for the first time in many foods. Its widespread occurrence in foods indicates that flazin is ingested daily during food consumption.

## 2 Materials and Methods

### 2.1 Foods and Chemicals

Food samples used for the analysis of flazin, including processed tomato and vegetable products, sauces, processed fruit products, beer, molasses, and honey (Table 1), were obtained in local supermarkets. Chemical compounds were obtained as follows: l-tryptophan (l-Trp), 5-(hydroxymethyl)furfural (5-HMF), and d-(−)-fructose from Sigma; d-(+)-glucose monohydrate from Merck (Darmstadt, Germany); 3-deoxy-d-glucosone (3-deoxyglucosone, 3-DG) from Biosynth-Carbosynth; and sucrose from Scharlau (Barcelona, Spain). Flazin was obtained and isolated from reactions of l-Trp and d-fructose, as mentioned below.

**Table 1:** Concentrations of the βC Flazin Found in Commercial Foods

foods	*X* (ng/g^a^ or ng/mL^b^)	SD	range (ng/g or ng/mL)
fried tomato (6)^c^	2086^a^	1105	126.8–3115
ketchup (3)	1938^a^	608.5	1245–2386
tomato juice (from concentrate) (4)	3200^b^	1918	1101–5603
concentrated tomato paste (3)	13944^a^	2357	12519–16664
tomato jam (2)	621.8^a^	321.2	394.7–849
soy sauce (4)	9401^b^	8901	2505–22390
barbecue sauce (2)	1040^b^	502.7	685–1396
balsamic vinegar (2)	35.4^b^	15.3	24.6–46.3
pineapple juice (from concentrate) (4)	269.6^b^	200.6	0–463
grape juice (from concentrate) (3)	70.73^b^	48.4	18.9–114.8
fruit juices (from concentrate) (3)^d^	86.06^b^	149.1	0–258.2
honey (4)	180.3^a^	146.0	40.0–384.8
beer (3)	96.03^b^	56.7	41.5–154.7
sugar cane molasses (1)	926.8^a^		
raisins (3)	460.7^a^	575.9	111.2–1125
dried prunes (2)	212.3^a^	231.7	48.5–376.1
dried apricot (1)	1677.2^a^		
plum jam (1)	245.9^a^		
fried onion (2)	165.0^a^	133.5	70.6–259.4

a ng/g.

b ng/mL.

c No. of samples.

d Tropical, pear, and multifruit juice.

### 2.2 Isolation, Purification, and Spectral Characterization of Flazin

Reactions of l-Trp (600 mg) and d-fructose (2400 mg) dissolved in 40 mL of 100 mM potassium phosphate buffer (pH 3) were carried out at 80 °C for 96 h and subsequently extracted with dichloromethane (150 mL) in alkaline pH (pH 9.5). The aqueous phase was taken to pH 2–3 and extracted with diethyl ether (150 mL). This organic phase was concentrated under a vacuum, redissolved into ethyl acetate (10 mL), and evaporated to get a crude solid that was subsequently purified by preparative HPLC. For that, a 1260 Infinity preparative HPLC Agilent apparatus with two pumps, a 1260 DAD detector, and an injection loop of 1.5 mL was used. For chromatographic separation, a 100 mm × 10 mm ACE 5 C18 column was used with 4.5 mL/min of flow rate and 0.1% formic acid (A) and 20% A in acetonitrile (B) as eluents with a linear gradient from 10% to 90% B in 15 min. Flazin eluted after 8.6 min. The isolated fraction was evaporated under vacuum to give flazin (1-[5-(hydroxymethyl)furan-2-yl]-9*H*-pyrido[3,4-*b*]indole-3-carboxylic acid or 1-(5-hydroxymethyl-2-furyl)-β-carboline-3-carboxylic acid) as a yellow powder (4.5 mg, 0.5% yield, 95% purity by HPLC) (Figure 1). Spectral characterization of flazin was accomplished by NMR experiments in a Varian NMR System.^40^ The resonance frequencies for ^1^H and ^13^C were 499.61 and 125.62 MHz, respectively, and spectra were recorded at 25 °C. Data were processed with the MestReNova software (version 14.3.3, Mestrelab Research SL, Santiago de Compostela, Spain). ^1^H NMR (500 MHz, DMSO-*d*_6_): δ 11.56 (s, 1H), 8.81 (s, 1H), 8.39 (d, *J* = 7.9 Hz, 1H), 7.82 (d, *J* = 8.2 Hz, 1H), 7.63 (t, *J* = 7.7 Hz, 1H), 7.41 (d, *J* = 3.2 Hz, 1H), 7.34 (t, *J* = 7.5 Hz, 1H), 6.61 (d, *J* = 3.3 Hz, 1H), 4.68 (s, 2H). ^13^C NMR (126 MHz, DMSO-*d*_6_): δ 166.83, 157.17, 151.21, 141.38, 131.67, 131.65, 129.81, 128.99, 128.72, 121.90, 120.98, 120.37, 115.39, 112.75, 110.82, 109.07, 55.91 (Table S1 and Figure S1). The NMR spectra of flazin agree well with the reported data of this substance.^41^ The high-resolution mass spectra (LC-MS-Q-TOF, Agilent) showed *m*/*z* 309.0966 [M + H]^+^, which corresponds to the C_17_H_13_N_2_O_4_ molecular formula and theoretical mass of 308.08753 amu (diff. 1.78 ppm). MS/MS mass spectrum (20 V): *m*/*z* 309 [M + H]^+^, 291 [M – H_2_O]^+^, and 263 [M-HCOOH]^+^.

### 2.3 Flazin in Reactions of l-Trp with 5-(Hydroxymethyl)furfural, 3-Deoxyglucosone, Carbohydrates, and Processed Foods

The formation of flazin was studied in model reactions of l-Trp with 5-HMF, 3-deoxyglucosone, and carbohydrates. Thus, reactions of l-Trp (0.5 mg/mL) with 5-HMF (0.1 mg/mL) or 3-deoxyglucosone (0.1 mg/mL) were performed in 100 mM potassium phosphate buffer (adjusted to pHs 1.3, 3.1, 5, 7.4, and 9) at 90 °C for 2–4 h and analyzed by HPLC. The reactions of l-Trp with 5-HMF or 3-deoxyglucosone (phosphate buffer, pH 3.1) were also carried out at temperatures ranging from 25 to 130 °C. Reactions of l-Trp and carbohydrates were performed as follows: (a) l-Trp (0.5 mg/mL) reacted with glucose (5 mg/mL), fructose (4.5 mg/mL), or sucrose (8.5 mg/mL) in potassium phosphate buffer (100 mM) (adjusted to pHs 1.3, 3.1, 5, 7.4, and 9) (20 h, 90 °C); (b) l-Trp (2 mg/mL) reacted with glucose (40 mg/mL), fructose (36.4 mg/mL), or sucrose (69.1 mg/mL) in potassium phosphate buffer (100 mM) (adjusted to pHs 3.1, 5, or 7.4) (80 °C, 20 h); (c) l-Trp (2 mg/mL) and fructose (36.4 mg/mL) in phosphate buffer (100 mM) (pH 2.9) reacted at temperatures from 37 to 130 °C for 20 h. All reactions were made in duplicate and were analyzed by HPLC-DAD/fluorescence and MS. The mechanism of formation of flazin was studied in reactions of l-Trp and 3-deoxyglucosone or fructose previously preheated at 100 °C (pH 3, 2–4 h) that reacted at 70 °C for 4 h and were analyzed by HPLC and HPLC-MS. The HPLC chromatographic peaks with an absorption maxima at around 355–375 nm (3,4-dihydro-β-carboline-3-carboxylic acids) (interval from 4.5 to 6 min) corresponding to these intermediates were collected in HPLC injections and evaporated to dryness at 45 °C under vacuum. They were redissolved in water, and aliquots (200 μL) were treated with the oxidant SeO_2_ (4 mg), adjusted pH to 3, then incubated at 70 °C for 3 h, and finally analyzed for flazin while comparing with corresponding controls. Flazin formation during processing was studied in several foods that were processed and compared to controls, as follows: (a) commercial fresh (not from concentrate) tomato juice heated (90 or 110 °C for 5 h); (b) fresh tomatoes crushed using an Ultraturrax homogenizer and heated (110 °C, 5 h); and (c) commercial canned natural crushed tomato puree heated (90 °C, 5 h).

### 2.4 Flazin Isolation by Solid Phase Extraction

Flazin in foods was isolated by solid phase extraction (SPE) with propylsulfonicacid-derivatized silica PRS cartridges (Bond Elut, 500 mg, 3 mL volume, Agilent). For that, samples of solid foods (2–5 g) or liquid foods (5 mL) were added to 0.6 M HClO_4_ (15–20 mL), homogenized with an Ultraturrax homogenizer, and centrifuged (12,000*g*, 5 °C) for 15 min. PRS cartridges were conditioned with 6 mL of methanol followed by 6 mL of 0.1 M HCl. Sample aliquots (5 mL) were spiked with 0.5 mL of 1-ethyl-β-carboline solution (EβC) (0.08 mg/L) as internal standard (IS) and loaded onto the PRS cartridges in a vacuum manifold. After washing with deionized water (2 mL), flazin was eluted with 3 mL of 0.4 M K_2_HPO_4_ (adjusted to pH 9.1), followed by 3 mL of 0.4 M K_2_HPO_4_ (pH 9.1)/methanol (1:1). These two phosphate fractions were mixed and subsequently analyzed by HPLC-fluorescence and HPLC-MS. The SPE procedure gave recoveries of flazin (80 μg/L) higher than 95% (*n* = 3) with a repeatability of 2.6% RSD (*n* = 3) and an accuracy of 8% mean error (*n* = 3).

### 2.5 HPLC and HPLC-MS Analysis of Flazin

The analysis of flazin in model reactions was performed on a 1050 HPLC instrument (Agilent Technologies) coupled to an 1100 series DAD and a 1046A fluorescence detector. The analysis of flazin in foods extracted with SPE was carried out in a 1200 series HPLC equipped with a 1200 series DAD and a 1260 series fluorescence detector (Agilent). For chromatographic separation, a 150 mm × 3.9 mm, 5 μm, Novapak C18 column (Waters) was used with 50 mM ammonium phosphate buffer adjusted to pH 3 (Eluent A) and 20% of eluent A in acetonitrile (Eluent B) using a gradient from 0 to 32% B in 8 min and 90% B at 12 min and with a flow rate of 1 mL/min, oven temperature of 40 °C, and injected volume of 20 μL. The concentration of flazin in the model reactions was obtained with a calibration curve of flazin standard detected with absorbance at 280 nm. The fluorescence conditions previously selected for perlolyrine^40^ were used for the analysis and detection of flazin in food extracts. Thus, fluorescence detection was carried out with excitation and emission programmed from 0 to 9 min at 300 nm (excit.) and 433 (emiss.) (detection of the IS) and changed to 420 nm (excit) and 460 nm (emiss.) at 9 min. The content of flazin was obtained from a calibration curve built with solutions of the flazin standard against EβC (IS), which were extracted following the SPE procedure. Identification of flazin was accomplished by HPLC with the spectra of DAD and fluorescence and by HPLC-MS. Food extracts from SPE were concentrated (45 °C) in a speedvac vacuum concentrator and analyzed by HPLC-MS (Electrospray ionization, ESI+).^32,40^ The apparatus was an HPLC-MS Waters separations module Alliance e2695 with a quadrupole QDa Acquity working under positive electrospray ionization (ESI+) (cone voltages at 10, 20, and 40 V) and with a C18 Atlantis T3, 2.1 mm × 100 mm (3 μm, 100 Å) column (Waters). The chromatographic conditions and mass spectra acquisition were the same as those used previously.^40^

## 3 Results

### 3.1 Isolation, Characterization, and Formation of Flazin

**Figure 2:**
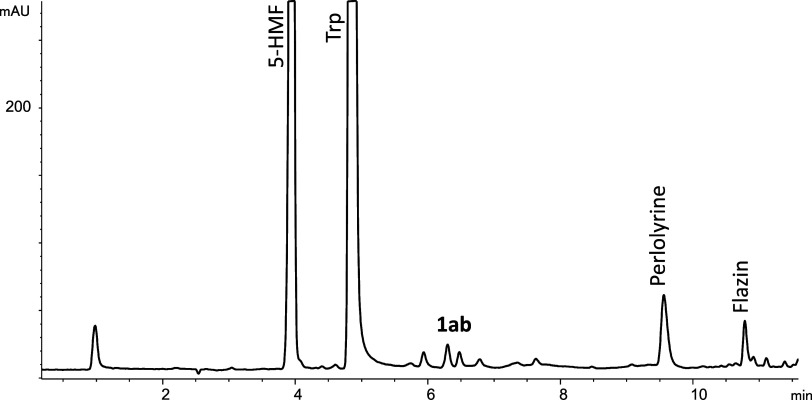
HPLC chromatogram (280 nm) of the reaction of 3-deoxyglucosone (0.1 mg/mL) with l-Trp (0.5 mg/mL) (90 °C, 4 h, pH 3.1) that gives flazin. Perlolyrine, **1ab** and 5-HMF are also formed.

**Figure 3:**
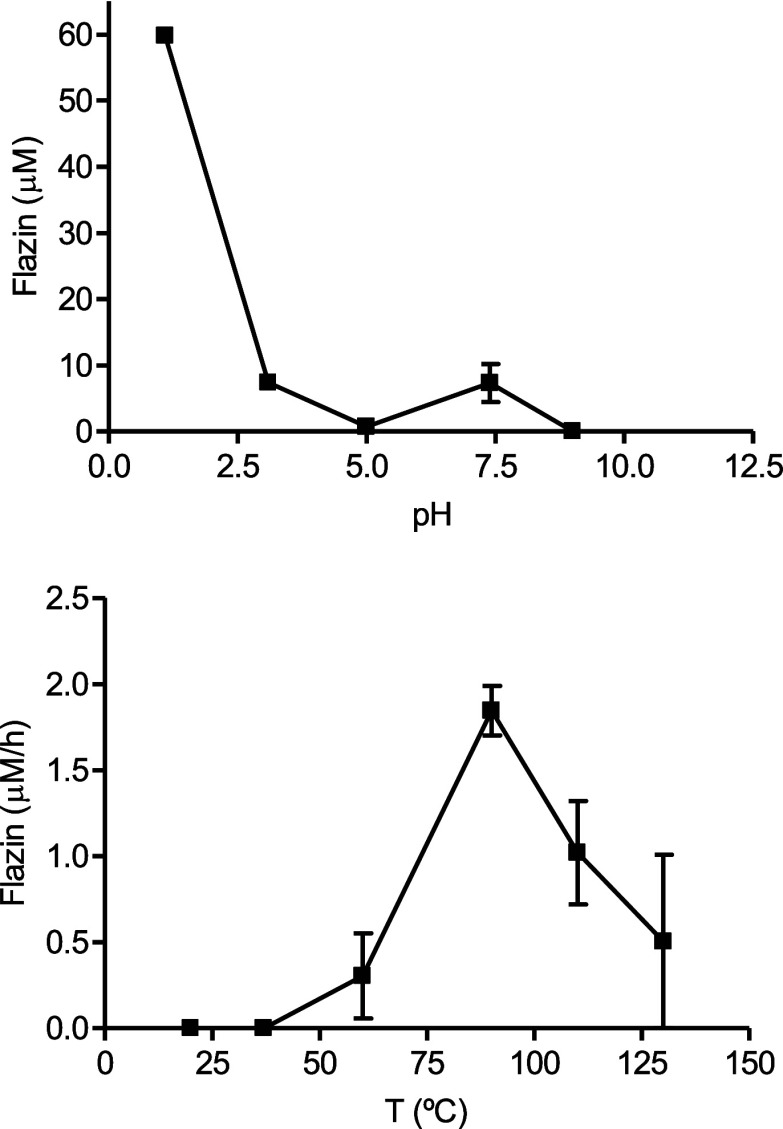
Formation of flazin from l-Trp (0.5 mg/mL) and 3-deoxyglucosone (0.1 mg/mL) as a function of pH (90 °C, 4 h) (a) and the formation rate of flazin with temperature (pH 3.1) (b).

Reactions of l-Trp with d-fructose (80 °C, pH 3.1) (Figure S2) were extracted in alkaline pH with dichloromethane, and subsequently, the aqueous phase was adjusted to pH 2–3 and extracted with diethyl ether. The ether fraction was evaporated to dryness, and a βC compound was isolated by preparative HPLC and characterized as flazin, which contains a hydroxymethylfuran moiety at C-1 and a COOH at C-3 (1-(5-hydroxymethyl-2′-furyl)-β-carboline-3-carboxylic acid) (Figure 1). Experiments with model reactions carried out in a range of pHs (1.3–9) and temperatures (25–110 °C) showed that flazin did not form under those conditions by Pictet–Spengler reactions from l-Trp and 5-HMF and oxidation. Instead, l-Trp did react with 3-deoxyglucosone and gave flazin (Figure 2). Flazin formation from 3-deoxyglucosone occurred simultaneously with other βCs such as the carbohydrate-derived βCs **1ab**(29,32) and perlolyrine^40^ (Figure 1). The formation of flazin from l-Trp and 3-deoxyglucosone with pH and temperature is illustrated in Figure 3. Flazin is highly increased under acidic pH and with increasing temperatures up to 90 °C. However, flazin was not favored at very high temperatures, such as 130 °C. Physiological conditions of pH and temperature (pH 7.4 and 37 °C) did not favor its formation.

**Figure 4:**
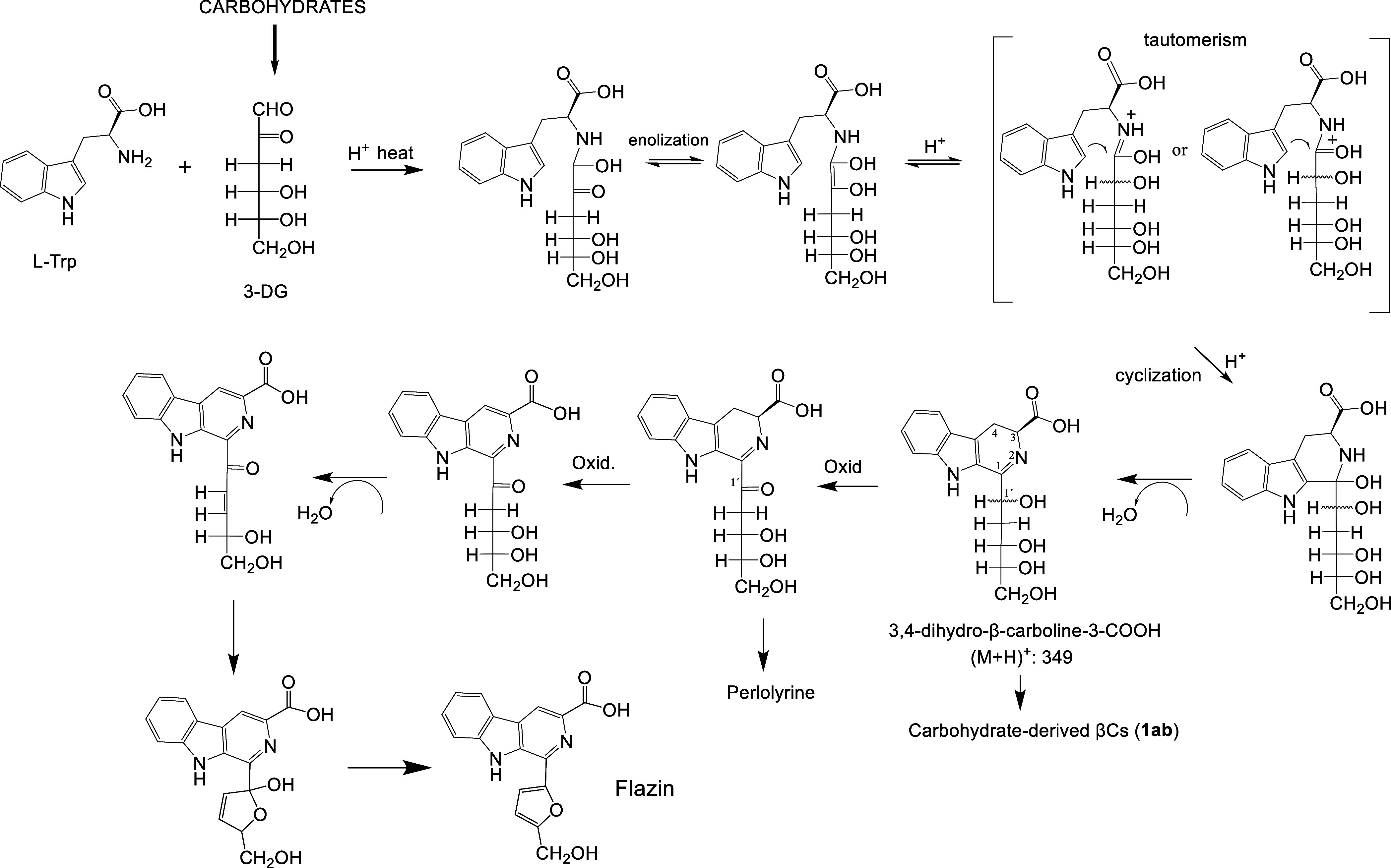
Proposed mechanism for the formation of flazin from l-Trp and 3-deoxyglucosone arising from carbohydrates.

The formation of flazin could follow the mechanism described in Figure 4. This mechanism is similar in the initial steps to that proposed for perlolyrine,^40^ and also for carbohydrate-derived βCs and α-dicarbonyl-derived βCs.^29,32^l-Trp reacts with 3-deoxyglucosone derived from carbohydrates, which following enolization, tautomerism, and cyclization affords intermediates 3,4-dihydro-β-carboline-3-carboxylic acid with C_1_′–OH in the carbohydrate chain. These dihydro-β-carbolines would be precursors of flazin. Indeed, they appeared at 70 °C and after a short time (4 h) in the reactions of l-Trp with 3-deoxyglucosone (or fructose preheated) and were detected by HPLC-MS ([M + H]^+^ at *m*/*z* 349; mass fragments at *m*/*z* 331, 285), and a λ_max_ at 355–375 nm in the absorbance spectra. It is proposed that the 3,4-dihydro-β-carboline-3-carboxylic acids (imines) with C_1_′–OH could be oxidized to C_1_′=O and aromatized (oxidized) to give the β-carboline-3-carboxylic acid that, following dehydration, cyclization, and another dehydration, will provide the furan ring of flazin (Figure 4). The same pathway with oxidative decarboxylation led to perlolyrine.^40^ This mechanism was supported by experimental results because the 3,4-dihydro-β-carboline-3-carboxylic acids with 355–375 nm of absorption maxima (HPLC fraction isolated at 4.7–6 min) gave flazin after oxidation with SeO_2_ and heating (Figure S3).

### 3.2 Formation, Identification, and Occurrence of Flazin in Reactions of l-Trp and Carbohydrates

**Figure 5:**
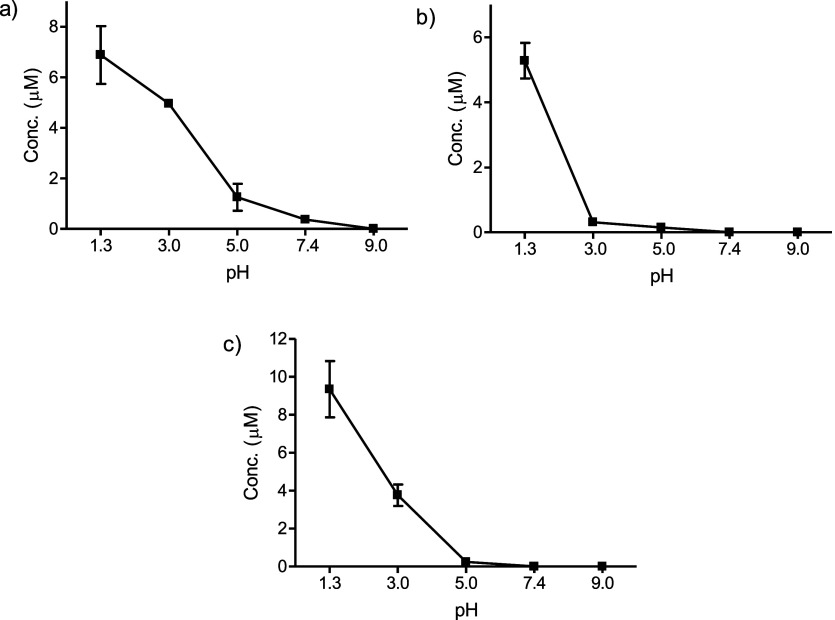
Formation of flazin from l-Trp (0.5 mg/mL) and fructose (4.5 mg/mL) (a), glucose (5 mg/mL) (b), and sucrose (8.5 mg/mL) (c) as a function of pH (90 °C, 20 h).

**Figure 6:**
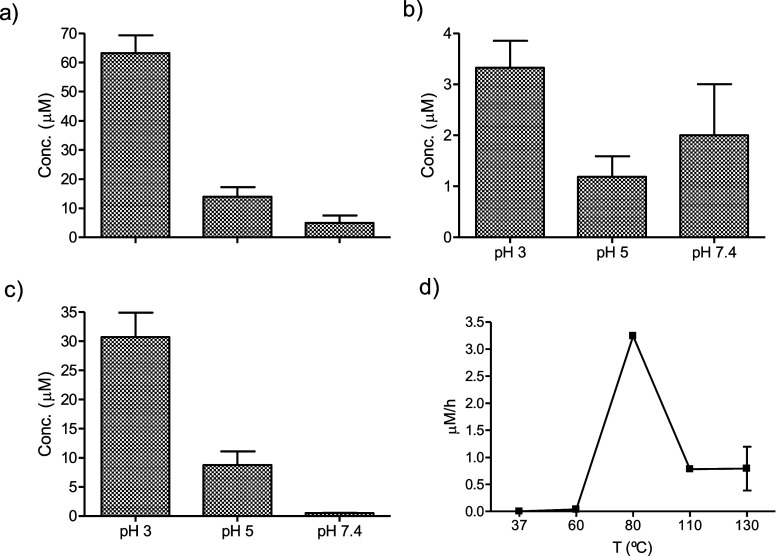
Formation of flazin in the reactions of high concentrations of l-Trp (2 mg/mL) and fructose (36.4 mg/mL) (a), glucose (40 mg/mL) (b) or sucrose (69.1 mg/mL) (c) (pH 3–7.4) at 80 °C for 20 h. Formation rate of flazin from l-Trp (2 mg/mL) and fructose (36.4 mg/mL) at different temperatures (pH 3) (d).

Flazin forms in the reactions of l-Trp with fructose, glucose, or sucrose incubated under heating (Figure S4). Its formation increased under acidic conditions (Figure 5). Flazin augmented in higher concentrations of l-Trp and carbohydrates, and it increased with temperature, although high temperatures (110–130 °C) resulted in a lower formation rate than 80 °C (Figure 6). Flazin increased under acidic pHs (e.g., pH 3), whereas formation at neutral pH (pH 7.4) was low. The formation of flazin produced from fructose was higher than that from glucose at pH 3 and higher. The formation of flazin arising from sucrose was similar to that from fructose under acidic conditions (e.g., pHs 1.3), but it decreased at higher pHs, indicating that sucrose hydrolyzes under acidic conditions and heating, and affords fructose that is involved in the formation of this βC. The formation of flazin from carbohydrates indicates that those are degraded under heating to 3-deoxyglucosone (3-DG) that reacts with l-Trp to afford flazin, as seen in Figure 4.

### 3.3 Flazin Identification and Occurrence in Foods

**Figure 7:**
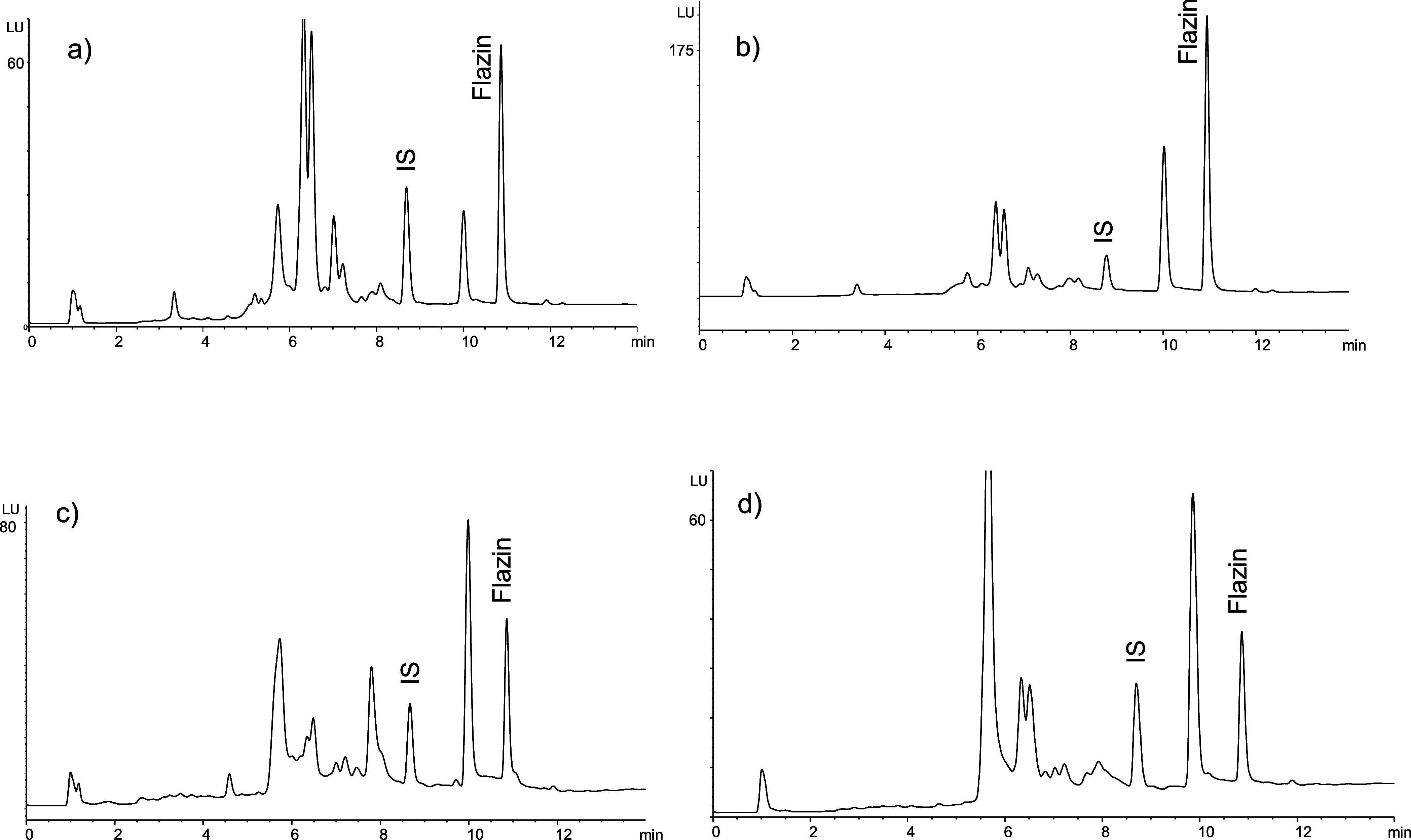
HPLC-FLD chromatograms of flazin in representative food samples analyzed following the isolation by SPE. (a) Tomato juice from concentrate, (b) tomato sauce ketchup, (c) toasted beer, and (d) pineapple juice. Fluorescence detection: 300 nm, excit. and 433 nm, emiss. (0–9 min); 420 nm, excit., and 460 nm, emiss. (9–14 min).

**Figure 8:**
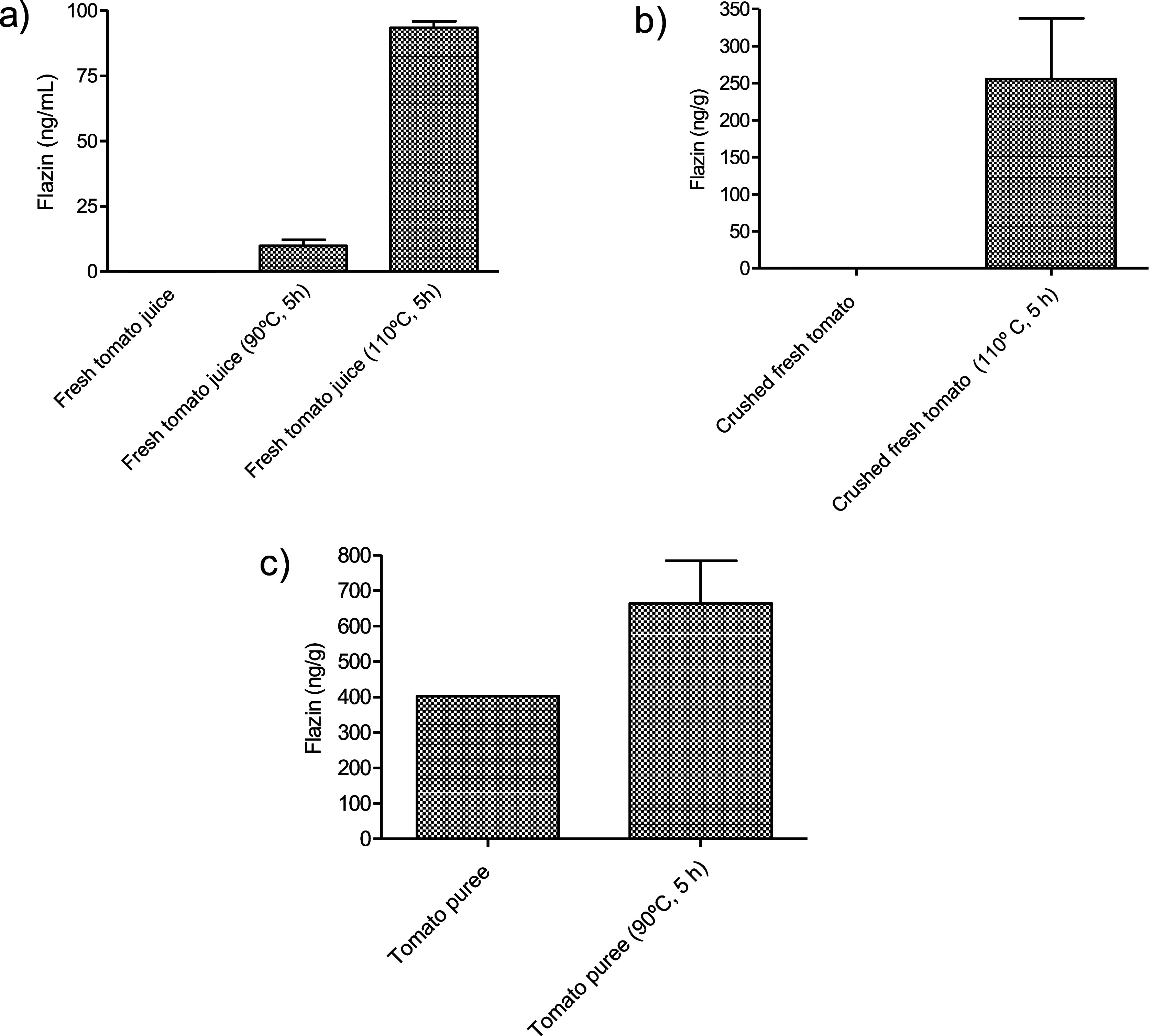
Formation of flazin in foods processed by heating. (a) Fresh tomato juice not from concentrate; (b) crushed fresh tomato, and (c) commercial canned crushed tomato puree and the same samples after heating in the laboratory.

The occurrence of flazin in foods was studied by HPLC-MS (Figure S5). It was present in many foods, including processed tomato products such as fried tomato puree, tomato juice, ketchup, tomato concentrate, and tomato jam, but also in sauces like soy sauce, sugar cane molasses, beer, fruit juices, and jams, as well as in dried fruits and honey. It was subsequently analyzed by HPLC-fluorescence detection (Figure 7), and the concentrations are listed in Table 1. Flazin appeared in many of the foods studied, and the highest levels were found in processed tomato products (fried tomato, ketchup, tomato juices, and tomato jam), with the highest mean level found in tomato concentrate (13,944 ng/g). A high level of flazin was also determined in soy sauce (9401 ng/mL). Flazin was found in relatively high levels in barbecue sauce, beer, and fruit juices elaborated from concentrate juice (grape and pineapple). Finally, flazin was also found in dried fruits (prunes and raisins), and fried onions, sugar cane molasses, balsamic vinegar, plum jam and honey. Other processed foods like cereals, breads, and cookies did not appear to contain flazin or had very low levels. These results suggested that flazin appeared in heat-processed foods. This fact was evidenced in samples of fresh tomato juice and crushed fresh tomatoes because flazin did not appear in fresh samples but increased by heating (Figure 8). In addition, a commercial sample of canned crushed tomato increased the flazin content after heating. This commercial sample already contained flazin, which was likely generated during the elaboration process.

## 4 Discussion

The results above have shown the isolation, characterization, and formation of flazin, a β-carboline-3-COOH that contains a furan ring and arises from the reaction of l-Trp and carbohydrates. Flazin is an analog of βC perlolyrine that exhibits interesting bioactive actions, including antioxidant and cytoprotection.^37,40^ As shown here, flazin was formed by the reaction of l-Trp with 3-deoxyglucosone, a product of the degradation of carbohydrates, and it appeared in the reactions of l-Trp with carbohydrates. Flazin yield increased under heating and acidic conditions. Very high temperatures (i.e., 130 °C) did not favor its formation compared to lower temperatures (e.g., 80–90 °C). A possible reason for that could be that other compounds derived from carbohydrates and 3-deoxyglucosone are favored at very high temperatures compared to flazin. Moreover, the formation of flazin under physiological conditions (37 °C, pH 7.4) was not favored; however, it was easily formed from l-Trp and carbohydrates with heating, evidencing that during food processing or cooking the formation of flazin can be remarkable.

The reaction of tryptophan with carbonyl compounds (e.g., formaldehyde and acetaldehyde) in foods affords tetrahydro-β-carboline-3-COOH (THβC-3-COOH) by the Pictet–Spengler reaction.^22,42,43^ The oxidation of THβC-3-COOH with or without decarboxylation converts these compounds into aromatic βCs (e.g., norharman and harman, βC-3-COOH).^26,27,44^ We studied whether the reaction of l-Trp with 5-hydroxymethylfurfural (5-HMF), a degradation product of sugars, affords THβC-3-COOH and the aromatic βC flazin by oxidation. However, flazin was not formed in that way. It is formed by the reaction of l-Trp with 3-deoxyglucosone (3-DG) in a reaction similar to that of other α-dicarbonyl- and carbohydrate-derived βCs^29,32^ as well as perlolyrine^40^ (Figure 4). The first steps of that reaction are similar to those reported first for βCs **1ab**, and analogous to βCs derived from α-dicarbonyl compounds.^29,32^ Thus, following enolization and tautomerism (keto–enol or imine–enamine), the cyclization affords 3,4-dihydro-β-carboline-3-carboxylic acid with an OH group at the C_1_′ position (C_1_′–OH).^29,32^ This mechanism is different from that of the Pictet–Spengler reaction since it led to 3,4-dihydro-β-carbolines instead of tetrahydro-β-carbolines. These imine intermediates could be oxidized to the corresponding C_1_′=O (ketoimines or alpha-iminoketones) under air or in the presence of oxidants,^45,46^ and further oxidized to the aromatic βC ring. Subsequently, this compound could dehydrate and cyclize through a reaction with C_4_′–OH to give a dihydrofuran ring and finally dehydrate again to give flazin (Figure 4). This mechanism was backed by results in this work. Thus, when the HPLC fraction (4.7–6 min) with 3,4-dihydro-β-carboline-3-carboxylic acids (with [M + H]^+^ ion at *m*/*z* 349 and with λ_max_ at 355–370 nm) was isolated and added with the oxidant SeO_2_ and heated, it gave flazin (Figure S3). Alternatively, the same pathway also produced perlolyrine by oxidative decarboxylation^40^ (Figure 4).

Flazin appeared in the reactions of carbohydrates with l-Trp under acidic conditions and heating. Flazin produced from fructose and sucrose after hydrolysis occurred in higher yields than that produced from glucose. The flazin precursor is the α-dicarbonyl compound 3-deoxyglucosone that is produced from carbohydrates, particularly fructose.^47−49^ 3-Deoxyglucosone is a predominant α-dicarbonyl compound in foods,^50^ it is also present in biological samples, including blood and plasma.^47,48^ The α-dicarbonyl derivatives react with free amino acids and proteins to give irreversible advanced glycation end products (AGEs), which may be involved in human diseases.^47,48,51−54^ As shown here, 3-deoxyglucosone reacts with l-Trp, affording flazin, which could be a new AGE product similar to other βCs derived from carbohydrates.^40^ The formation of flazin is not favored under normal physiological conditions, but it is easily produced in food processing and cooking. Therefore, flazin is daily uptaken via food ingestion and could appear in the body as occurs with other βCs.^1^

The presence of flazin in many commercially processed foods was evidenced following its isolation by SPE and HPLC-MS. Those included concentrate tomato paste, tomato juice (from concentrate), fried tomato paste, tomato sauces and ketchup, tomato jam, beer, sauces like soy sauce, fruit juices, dried fruits (raisins, prunes, and apricots), fruit jam, honey, and molasses (Table 1). The flazin content varied among foods but also between samples within the same type of food, suggesting the importance of the elaboration process. The highest concentration was found in tomato processed products and soy sauce, followed by fruit juices from concentrate, dried fruits, beer, and honey. Flazin was formed during food processing by heating, as shown in fresh tomato juice and tomato puree (Figure 8). Therefore, foods containing l-Trp and carbohydrates in an acidic environment and processed by heating will generate flazin. The processing conditions will likely determine the level of flazin in food samples. Our former knowledge about flazin in foods was scarce, whereas the factors and mechanisms involved in its formation remained unknown. Flazin was previously identified in soy sauce^35,36,55^ and recently in tomato juice,^56,57^ but its presence and levels in most foods remained unknown. Remarkably, the levels of flazin measured here are generally higher than those of perlolyrine, which is its corresponding decarboxylated βC,^40^ and also higher than βCs **1–3** also arising from 3-deoxyglucosone.^29,32^ Indeed, these βCs have the same precursor and occur in the same foods (e.g., processed tomato products). A higher formation of flazin over perlolyrine in foods (e.g., tomato products and others) suggests that the formation (oxidation) from the intermediate precursors (Figure 4) preferably occurs without decarboxylation. The amounts of flazin in foods were also higher than those of the βCs from methylglyoxal and glyoxal,^32^ and higher than harman and norharman.^44^ Taking together, the wide presence of this βC in commercial foods, along with its easy formation during food processing/cooking, indicates that flazin is ingested daily via food uptake. An estimated exposure could reach up to hundreds of μg of flazin/person per day. In addition, the consumption of natural products might enhance the ingested flazin. Flazin has been previously identified in extracts of *Nitraria tangutorum* fruit,^58^ the seeds of *Brucea javanica,*(59) and *Crassostrea* oysters.^39,60^ Flazin in those products might have been formed by a reaction of carbohydrates (3-deoxyglucosone) with l-Trp, as shown here.

The βC alkaloids are considered bioactive substances that inhibit key enzymes (e.g., MAO and DYRK1 kinases), bind to CNS receptors, and exert other biological activities.^1,2,16−19^ Some of these compounds inhibit MAO enzymes, showing antidepressant and neuroprotective actions.^9−14,16^ The βCs harman and norharman, which appear in foods, are good inhibitors of MAO^29,61^ and are antioxidants against free radicals.^18^ The βCs can be comutagenic compounds and could be bioactivated in vivo into neurotoxic β-carbolinium cations.^3,27,62,63^ The furan-βC perlolyrine (Figure 1) increased phase II enzymes like quinone reductase (QR), which are chemoprotective.^36,38^ Perlolyrine exerted antiproliferative actions in vitro against tumor cells at micromolar levels^37^ and activated the human vanilloid TRPV1 and ankyrin (TRPA1) receptors.^64^ Flazin was also an inducer of the QR enzyme at micromolar concentrations^36^ and showed antioxidant effects in vitro at micromolar levels through the activation of the Keap1-Nrf2 system, resulting in cytoprotection.^38^ It inhibited glycation,^57^ ameliorated lipid droplet accumulation,^39^ and showed immunomodulatory^60^ and anticarcinogenic effects.^55,59^ Therefore, the furan-βCs are bioactive compounds, but additional research is needed to clarify their actions. The widespread presence of flazin in foods, as shown here, suggests that it will be daily ingested, and potentially, it could exert bioactive actions in the body. Moreover, the βCs derived from α-dicarbonyls like flazin are new AGEs products.^29,32^ Although flazin formation is not favored under physiological conditions, its formation in foods, food processing, and cooking could result in a decrease of 3-deoxyglucosone, which is ultimately involved in glycation.

Taken this together, it is concluded that 3-deoxyglucosone reacts with tryptophan, giving flazin, which is a bioactive β-carboline-3-carboxylic acid with a furan moiety. The proposed mechanism of formation occurs through the formation of 3,4-dihydro-β-carboline-3-carboxylic acid, which is oxidized to α-ketoimine (C_1_′=O) and aromatic βC without decarboxylation. Those intermediates can suffer dehydration, cyclization to the dihydrofuran ring, and another dehydration to give flazin. Flazin formation was favored under heating and acidic conditions. The optimal temperature was about 80–90 °C, whereas high temperatures (130 °C) decreased formation rates. Flazin appeared in the reactions of tryptophan with carbohydrates under acidic conditions and with heating. Flazin produced from fructose was much higher than that formed from glucose, whereas sucrose produced flazin after acidic hydrolysis and heating. Flazin appeared in many processed foods, and among them, tomato products and soy sauce contained the highest concentrations. It was produced during the heating process. Flazin is a bioactive βC alkaloid with antioxidant and chemoprotective properties. These results indicate that flazin is ingested daily due to its presence in foods, and its exposure could increase with food cooking.

## Supporting Information Available

Supporting Information includes Table S1 and Figures S1–S5 containing the NMR signals of flazin and HPLC and HPLC-MS chromatograms of flazin in the reactions and foods (PDF)
