# Supplementary material for: Formation, Identification, and Occurrence of the Furan-Containing β-Carboline Flazin Derived from l-Tryptophan and Carbohydrates
Source: J Agric Food Chem. 2024 Mar 12;72(12):6575–84. doi: 10.1021/acs.jafc.3c07773 (PMC10979450; doi:10.1021/acs.jafc.3c07773)
Supplement: Supplementary file 1 — jf3c07773_si_001.pdf [file jf3c07773_si_001.pdf]

Supporting information

FORMATION, IDENTIFICATION AND OCCURRENCE OF THE FURAN-CONTAINING β-CARBOLINE FLAZIN DERIVED FROM L-TRYPTOPHAN AND CARBOHYDRATES

Tomás Herraiz[1]* and Antonio Salgado[2]

[1]Instituto de Ciencia y Tecnología de Alimentos y Nutrición (ICTAN-CSIC). Spanish National Research Council (CSIC). José Antonio Novais 6, Ciudad Universitaria 28040, Madrid (Spain).
[2]Centro de Espectroscopía de RMN (CERMN), Universidad de Alcalá (UAH), Campus Universitario Ctra. Madrid-Barcelona km 33.6, 28805, Alcalá de Henares, Madrid (Spain).

Flazin NMR data

General experimental procedures.

NMR experiments of flazin were done in a Varian NMR System (Varian Inc, Palo Alto, CA, USA), equipped with a CHX $^{1}H/^{13}C/^{15}N-^{31}P$ probe head, a z -gradient module and a variable temperature unit. The resonance frequencies for ^{1}H and ^{13}C were 499.61 and 125.62 MHz, respectively. All spectra were recorded at 25ºC. NMR raw data was processed with the MestReNova software (version 14.3.3, Mestrelab Research SL, Santiago de Compostela, Spain).

^{1}H NMR (500 MHz, DMSO-d_6) δ 11.56 (s, 1H), 8.81 (s, 1H), 8.39 (d, *J* = 7.9 Hz, 1H), 7.82 (d, *J* = 8.2 Hz, 1H), 7.63 (t, *J* = 7.7 Hz, 1H), 7.41 (d, *J* = 3.2 Hz, 1H), 7.34 (t, *J* = 7.5 Hz, 1H), 6.61 (d, *J* = 3.3 Hz, 1H), 4.68 (s, 2H).

^{13}C NMR (126 MHz, DMSO-d_6) δ 166.83, 157.17, 151.21, 141.38, 131.67, 131.65, 129.81, 128.99, 128.72, 121.90, 120.98, 120.37, 115.39, 112.75, 110.82, 109.07, 55.91.

Table S1: ^{1}H NMR and ^{13}C NMR of flazin

	**^{13}C δ (ppm)**	**^{1}H δ (ppm)**
C-1	129.81	-
N-2	-	-
C-3	131.65 or 131.67	-
C-4	115.39	8.81 (s, 1H)
C-4a	128.99	-
C-4b	120.98	-
C-5	121.90	8.39 (d, *J* = 7.9 Hz, 1H)
C-6	120.37	7.34 (t, *J* = 7.5 Hz, 1H)
C-7	128.72	7.63 (t, *J* = 7.7 Hz, 1H)
C-8	112.75	7.82 (d, *J* = 8.2 Hz, 1H)
C-8a	141.38	-
NH	-	11.56 (s, 1H)
C-9a	131.65 or 131.67	-
furan C-2	157.17	-
furan C-3	109.07	6.61 (d, *J* = 3.3 Hz, 1H)
furan C-4	110.82	7.41 (d, *J* = 3.2 Hz, 1H)
furan C-5	151.21	-
3-CO_2H	166.83	n. d.
CH_2OH	55.91	4.68 (s, 2H)
$CH_2O\underline{H}$	-	n. d.

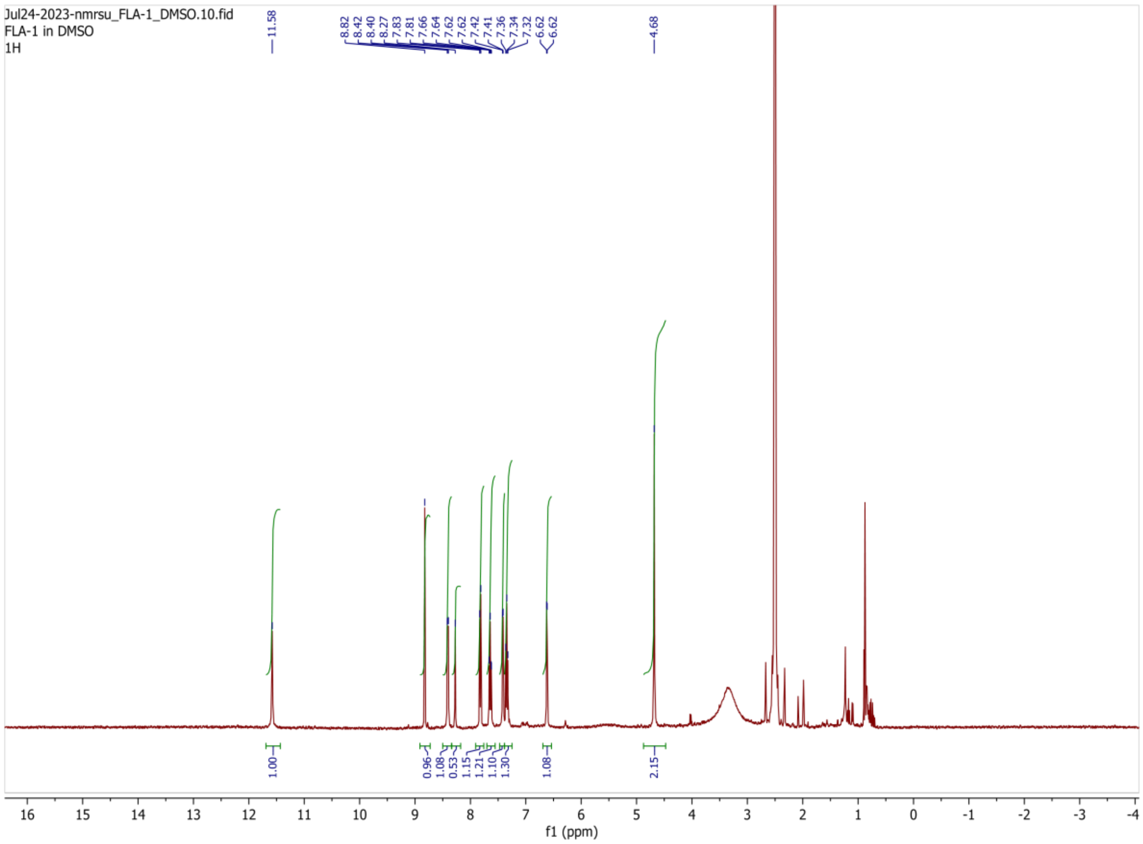

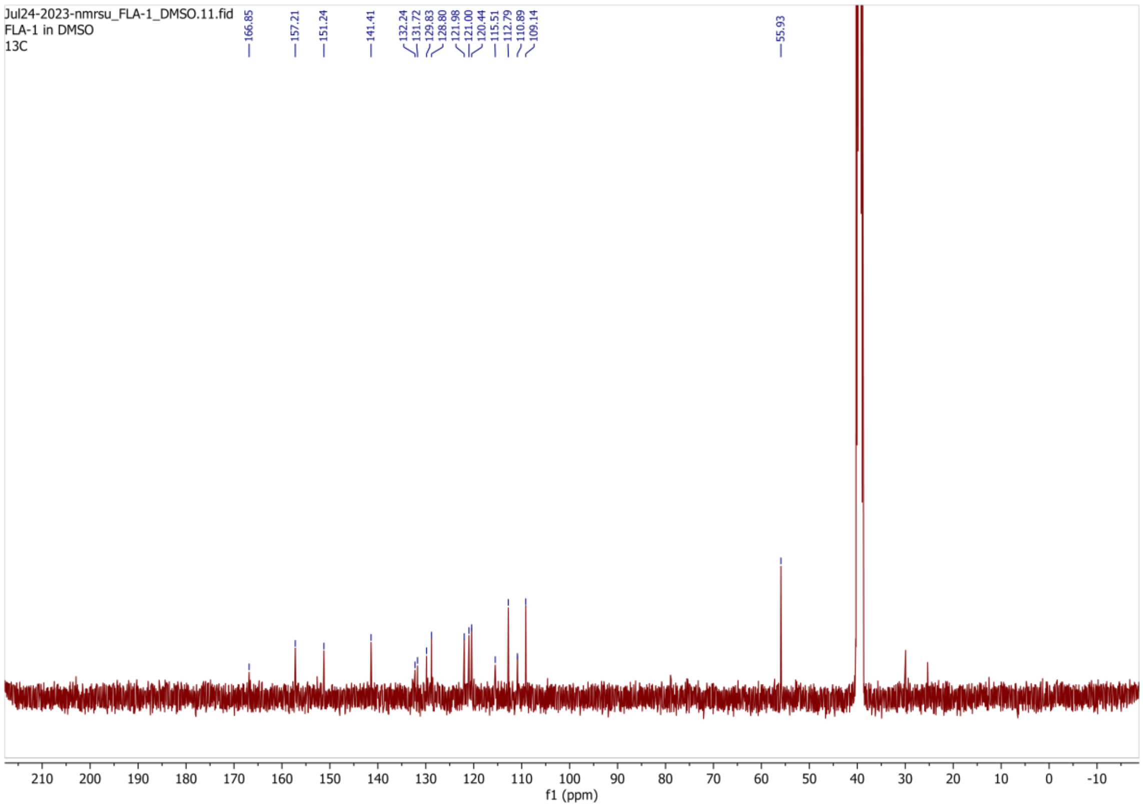

Figure S1: ^{1}H NMR and ^{13}C NMR spectra of flazin

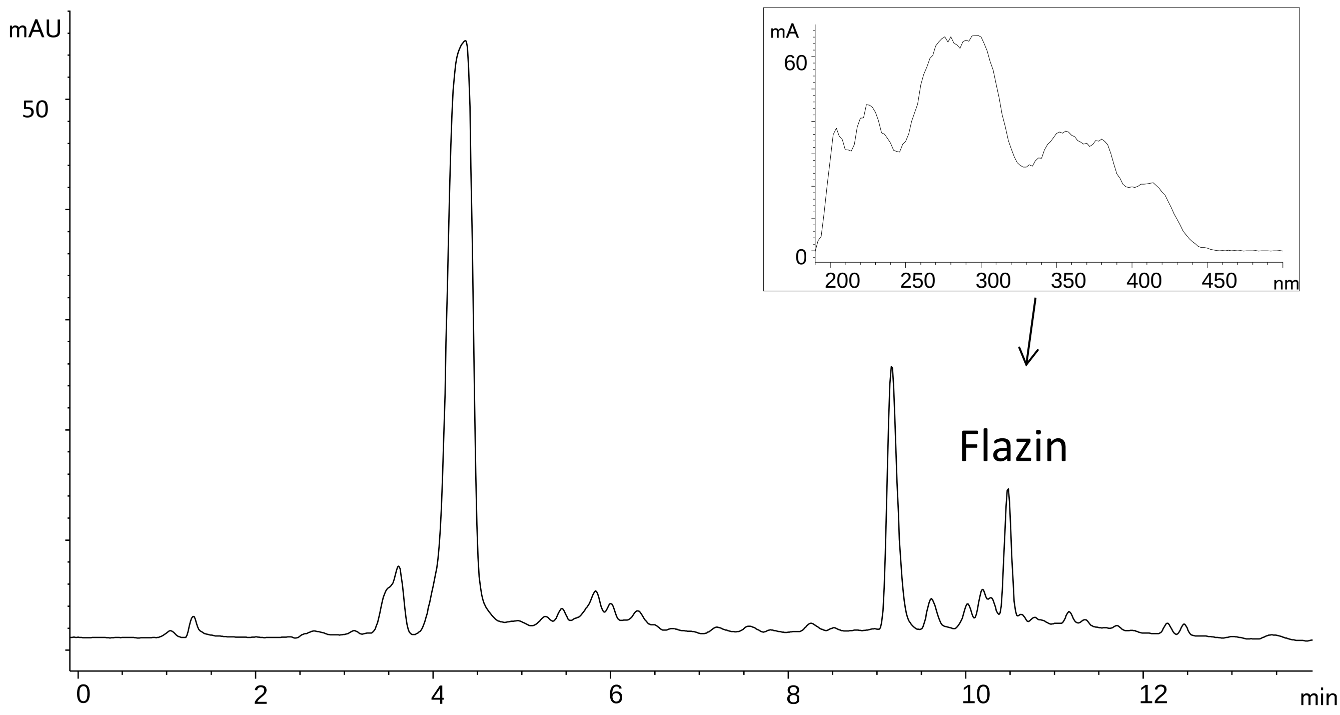

Figure S2. HPLC chromtagram of the mixture of tryptophan and D-fructosa reacted at pH 3, 80ºC from where flazin was isolated.

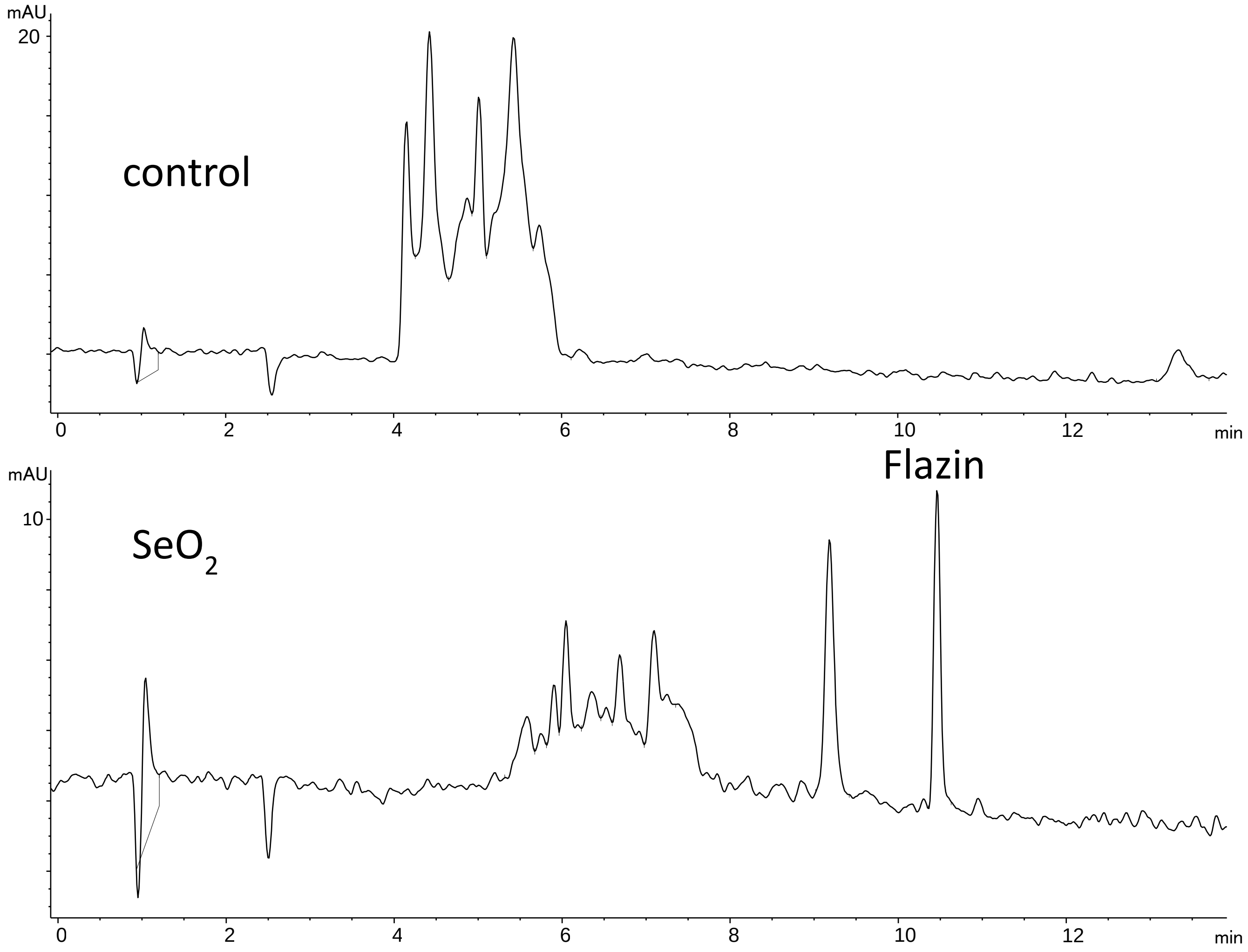

Figure S3. Formation of flazin from intermediates isolated from the reaction of tryptophan and fructose (preheated at 100 ºC). a) control and b) formation of flazin following treatment with SeO_2 and heating.

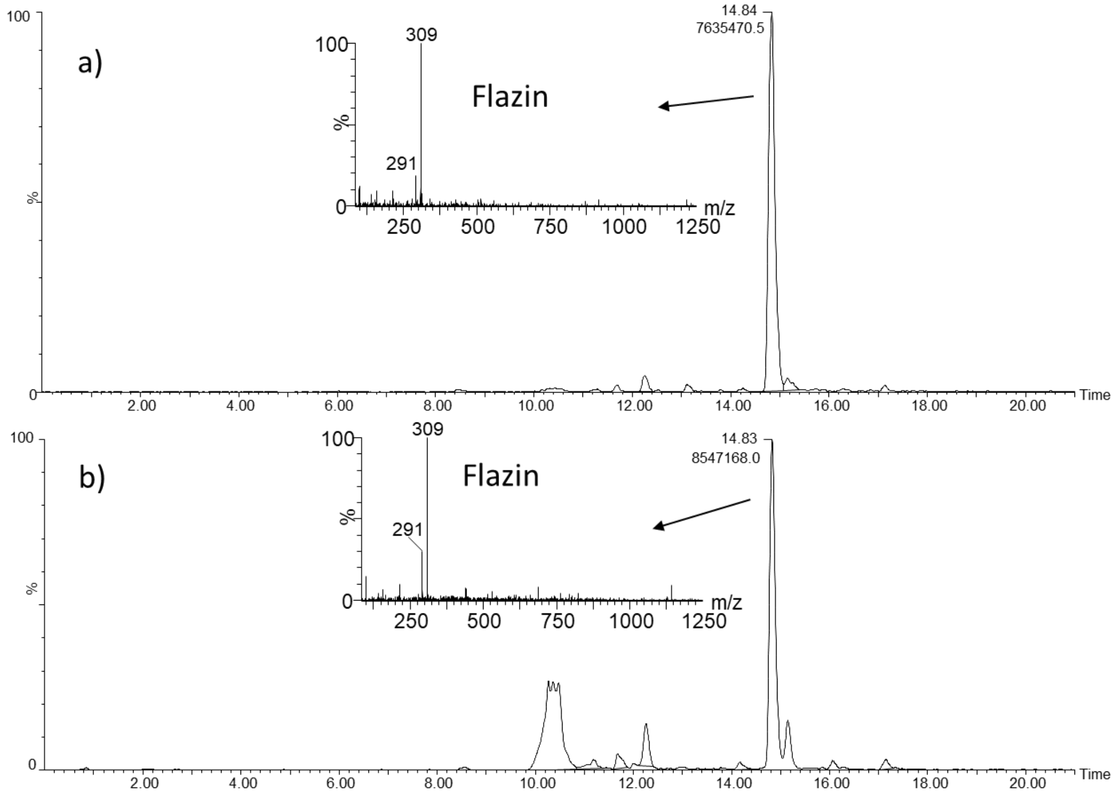

Figure S4. HPLC-MS chromatogram of the reaction of trp (0.5 mg/mL) with glucose (5 mg/mL) (a) or fructose (4.5 mg/mL) (b) (pH1.3, 80ºC, 20 h).

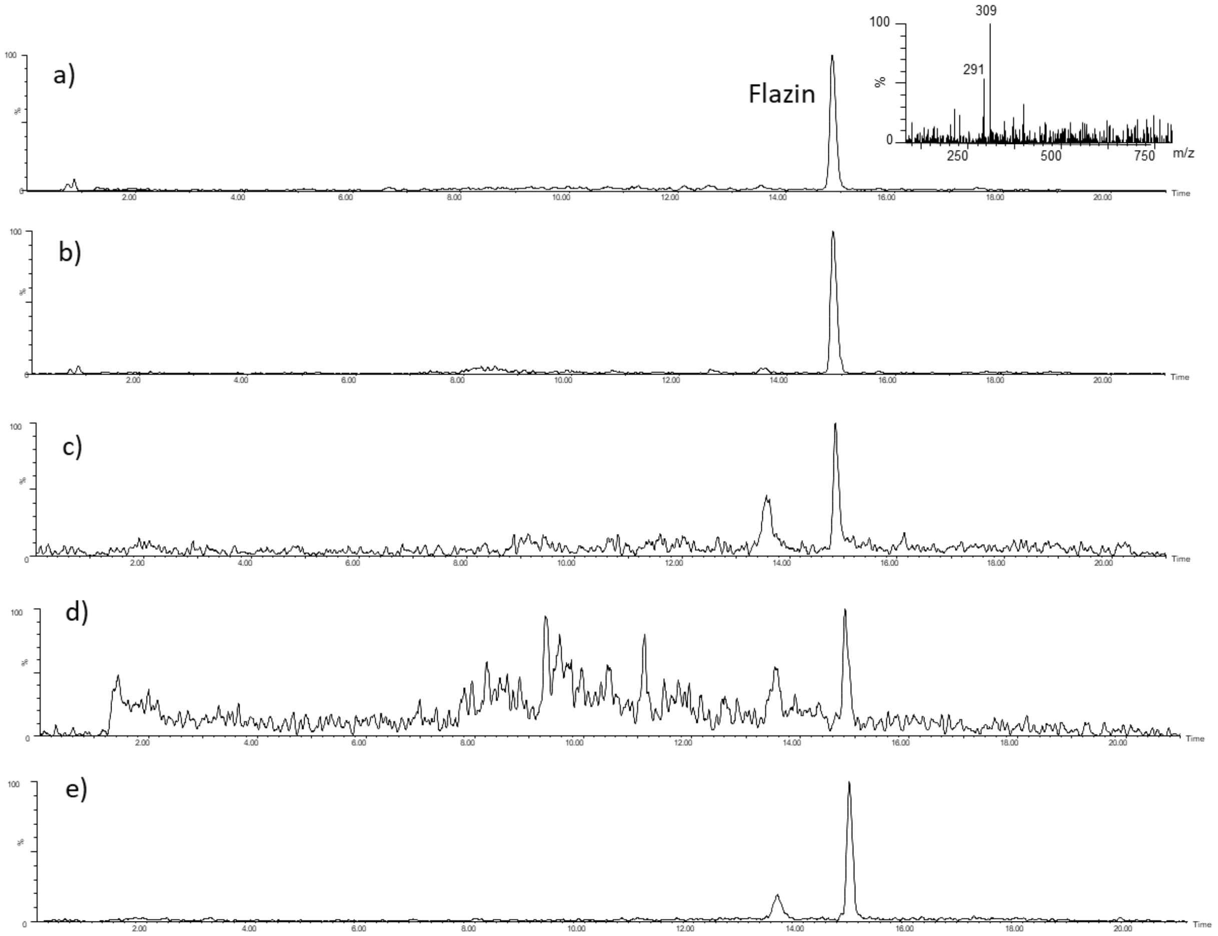

Figure S5. Chromatoagram of HPLC-MS on the presence of flazin in food samples: a) tomate concentrate paste, b) ketchup, c) dried prunes, d) toasted beer and e) sugarcane molasses.
